# Self-Reported Coping Strategies for Managing Work-Related Stress among Public Safety Personnel

**DOI:** 10.3390/ijerph19042355

**Published:** 2022-02-18

**Authors:** Gregory S. Anderson, Rosemary Ricciardelli, Linna Tam-Seto, Sulaimon Giwa, R. Nicholas Carleton

**Affiliations:** 1 Faculty of Science, Thompson Rivers University, Kamloops, BC V2C 0C8, Canada; ganderson@tru.ca; 2 Department of Sociology, Memorial University of Newfoundland, Saint John’s, NL A1C 5S7, Canada; 3 Department of Psychiatry and Behavioural Neurosciences, McMaster University, Hamilton, ON L8S 4L6, Canada; tamsetol@mcmaster.ca; 4 School of Social Work, Memorial University of Newfoundland, Saint John’s, NL A1C 5S7, Canada; sgiwa@mun.ca; 5 Department of Psychology, University of Regina, Regina, SK S4S 0A2, Canada; nick.carleton@uregina.ca

**Keywords:** coping, public safety personnel, stress, mental health, injury

## Abstract

Public safety personnel (PSP) experience a disproportionately high number of on-the-job stressors compared to the general population. PSP develop self-initiated actions, or coping strategies, that either confront the situation (approach strategies) or avoid the situation (avoidance strategies) to reduce the impact of stressors on their well-being. Understanding how PSP cope with stress is critical to ensuring their safety and that of the public. In the current study, we examined the coping strategies of PSP (*n* = 828 in the total sample). Participants managed their experiences of occupational stress or distress using three primary approach coping strategies: education (learning about mental illness and their causes), self-reliance (processes of self-reflection), and treatment (pharmaceutical, psychotherapy) that were considered adaptive. Results demonstrate PSP used multiple coping strategies simultaneously to deal with occupational stress. PSP who reported doing better tended to attribute their success to treatment, specifically psychotherapy, either alone or in combination with other interventions, and almost always emphasizing important supports from co-workers, families, and friends. Changing workplace culture could help to de-pathologize the effects of stress reactions being perceived as individual “failings”.

## 1. Introduction

Public safety personnel (PSP) are professionals frequently called upon to provide services, often during emergencies or disasters, to protect life and property, and to maintain safety and order [1,2]. Such professionals include, but are not limited to, correctional workers, firefighters, paramedics, police, and communication officials [3]. PSP are unified in a mandate to protect people from loss and suffering, which often results in significant physical and emotional demands. PSP work in high-stress, emotionally charged, and psychologically demanding jobs [1,4,5]. PSP also have disproportionately high exposure rates to potentially psychologically traumatic events (PPTE), which include “direct or indirect experiences of actual or threatened death, serious injury, or sexual violence” [6] (p. 38), which increases risk for physical and mental health injuries.

Repeated exposure to PPTE increases risk for the development of work-related health issues among employees. Sterud, Ekeberg and Hem (2006) reported the prevalence of paramedic posttraumatic stress disorder (PTSD) at greater than 20% [7]. A diverse sample of rescue workers evidenced a pooled prevalence of PTSD of 10%, with the highest prevalence in paramedics (14.6%) [8]. Recent results of a national survey examining prevalence of symptoms consistent with mental disorders among PSP in Canada report higher prevalence of mental disorders with 44.5% of respondents screening positive for clinically significant symptoms of one or more mental disorders such as PTSD and major depressive disorder [3]. In any case, the prevalence rates for PSP appear much higher than the 10% reported for the general population [9]. Carleton and colleagues also found a substantial rate of PSP report high past year and lifetime suicidal ideation (10.1% to 27.8%), planning (4.1% vs. 13.3%), or attempts (0.4% vs. 4.6%) [10]. The same study highlighted that among PSP, women were at higher risk for lifetime suicidal behaviours than men (ORs = 1.15 to 2.62), suggesting gender needs to be considered in reactions to, and coping strategies for, work stress [10]. The impact of repeated exposures to PPTE is evident in a systematic review of the literature [7]; specifically, PSP who retired on medical grounds report increased prevalence of PTSD symptoms and anxiety cumulating in early retirement. Significant occupational stress and PTSD have each been associated with work-related performance decrements, individual functional impairments in complex environments, and reduced quality of life [11,12].

Donnellan and colleagues (2006) posit stress as the transaction between the stressor and an individual’s cognitive construction (e.g., based on their appraisal) of the stressor [13]. Primary appraisal of stress involves perceiving a factor or incident as stressful or not, whereas secondary appraisal concerns perceptions of agency and capacity to cope with a stressful factor or incident [14]. Accordingly, effective coping depends on the goodness of fit between a perceived stressor and an individual’s coping strategy, as mediated by personal appraisal of the stressful event [4,15].

Personal coping strategies are central to the self-care of PSP who have frequent PPTE exposures [16]. PSP can develop coping strategies to help them recognize and process physical or psychological stressors that cause distress requiring the use of personal resources and attention [14,17]. Lazarus and Folkman (1984) describe problem or task-focused coping (e.g., active coping, planning, acceptance, positive re-framing) and emotion-focused coping (e.g., peer and emotional support, venting, engagement, humor), as underpinning current conceptualizations of approach coping strategies [17,18]. The concept of avoidance coping was introduced later [19,20] to capture behaviours designed to distance a person from a PPTE and the associated emotional tension (e.g., excessive alcohol use, withdrawal, denial).

Researchers often operationalize coping research by examining various coping strategies (i.e., behaviours, skills, or ways of regulating thoughts and emotions) that enable recognizing and responding to operational and organizational stressors [21]. Contemporary definitions of coping strategies describe a suite of self-regulatory techniques that either confront the situation or stressor (approach strategies) or avoid the situation or stressor (avoidance strategies) [22]. There are many terms used in the literature to describe approach and avoidance strategies. Approach coping terms can include positive, adaptive, engagement, and approach coping; avoidance coping terms can include negative, maladaptive, disengagement, and avoidant coping [15,21]. 

A simple binary categorization of coping strategies over-simplifies exceptionally complex behaviours, some of which could be framed as approach or avoidance coping; nevertheless, the heuristic offers a useful heuristic for researching PSP coping strategies. Approach coping strategies generally involve directly engaging with emotions, memories, and physical sensations to facilitate more adaptive stress management [23,24]. Avoidance coping strategies generally involve active distancing from stressful stimuli, and have been associated with poorer mental health [15] and increased pathology [24]. Approach coping does not always enhance positive affect and avoidance coping may provide necessary time for recovery [25,26]. PSP who seek social support or engage in adaptive emotion regulation techniques (e.g., acceptance, positive reframing) demonstrate approach coping [27]. PSP who withdraw or become disengaged, or who actively use denial, wishful thinking, suppression of either emotion or thought, or substance use demonstrate avoidance coping [27]. The impact of using any coping strategy is contingent on the situation, individual capacities to use the strategy to manage a stressor [17,28,29], and individual mental health and well-being [30]; however, approach coping strategies are typically more effective for stress management than avoidance coping strategies, and can be heuristically described as adaptive coping strategies for the current study [30].

Despite growing interest in supporting PSP mental health and well-being, there is very little research on the strategies PSP use to cope with PPTEs. A recent structured review and meta-analysis [21] found no clear evidence of a universally effective approach to develop or enhance adaptive coping strategies in PSP populations. Indeed, there are also very few measurement tools specific to PSP coping and mental health [31]. Di Nota et al., 2021 concluded there is “an urgent need to identify effective and adaptive coping strategies that can effectively mitigate the adverse psychological effects of traumatic occupational exposures” [20] (p. 14). The current study was designed to explore the coping strategies identified by participants from a large sample of Canadian PSP of which 44.5% screened positive for one or more mental health disorders [3]. The current study focused on identifying adaptive coping strategies PSP report using in response to repeated exposures to occupational (i.e., operational and organizational) stressors. 

## 2. Materials and Methods

The current qualitative study draws on data from a national survey that examined the prevalence of mental health disorders among PSP (see [3]). 

### 2.1. Participants 

All participants provided online consent prior to the completion of the online survey examining the mental health and well-being of a cross section of Canadian PSP. A total of 9260 PSP began the bilingual (English or French) online survey of which 828 participants elected to provide final comments in an open-ended text space to the statement: “If you have any additional information you would like to provide or additional feedback, please feel free to do so below.” The subset of those responding to the open-ended question were the participants in the analyses and were all active employees (i.e., not on sick leave) and included persons reporting having had poor mental health, were undergoing a change or intervention of some form, and were now “doing much better” or working towards “doing better.” Comments included in the current analyses were directly quoted by participants and were explicitly approved for use in the current study. There were 269 female and 556 male participants, and 3 who declined to report their sex. Most participants were geographically located in the Western provinces (i.e., British Columbia, Alberta, Saskatchewan, and Manitoba; *n* = 433), about a quarter in Ontario (*n* = 233), and the remainder in Quebec and the Atlantic provinces (*n* = 148). A range of occupational groups were represented within the sample, with the largest representation coming from RCMP, paramedicine, municipal police, firefighters, and operational correctional workers. Other groups with smaller representation included provincial police, communication specialists, and Canadian Border Services (see Table 1).

### 2.2. Procedure

The current data were collected using a secured web-based self-report survey following established guidelines for web-based surveys [32]. PSP were recruited through public safety organizations by means of social media, organizational websites, unions, and emails sent via employers, professional associations, and researchers as well as organizational listservs. The former Minister of Public Safety and Emergency Preparedness in Canada also provided support for the survey through an online video that served to assist with recruitment. The survey issued each participant a unique computer-generated random code that allowed for repeated non-duplicate entries, therein accommodating PSP schedules and facilitating participation. The study received approval from the University of Regina Institutional Research Ethics Board (File No. 2016-107). 

### 2.3. Data Analyses 

Effective and adaptive coping strategies were identified by focusing the current work on PSP who also self-reported having better mental health. Thematic inductive qualitative analyses were used to analyze the data, identifying and drawing on emergent themes [33], while incorporating a constructed semi-grounded approach [34]. First, participant responses, which ranged in length from a sentence to multiple paragraphs, were imported from the Qualtrics database to an Excel spreadsheet for import into QRS NVivo Pro (QSR International, Burlington, MA, USA) From there, responses were autocoded as unique cases along with their attributes. The autocode function allows for a set of “Attributes” to be assigned, which ensures that the data was labeled in a way that gender and occupation could be controlled for during analyses. An attribute is the process of assigning and managing the demographic information (e.g., gender, race) of respondents or cases. The autocode function enabled the classification of respondents’ data into ‘nodes,’ to facilitate the identification of preliminary patterns and ideas, and later ‘child nodes.’ Using autocode functions, researchers completed multiple reviews of the data to become fully immersed and to gain a sense of the data as a whole [35]. The first 100 responses were then read and categorized as themes (nodes). Using the constructed semi-grounded approach, all responses that shared similar ideas (i.e., social support, family, structured support, and self-care) were grouped together, to develop a preliminary coding scheme. A more detailed review followed, where secondary codes (themes, child nodes) were identified throughout each primary code [33]. The analytic process allowed for nodes to be restructured, producing a complete and cohesive picture of the data [36]. An exhaustive list of themes was created through repeated re-reading of transcripts and axial coding [35]. All the information within each of the categories was coded (see Table 2) and then analyzed to present the aggregate of emergent themes within the context of PSP coping practices. 

## 3. Results

The current results focused on approach coping strategies to identify adaptive coping strategies that may help to improve overall mental health and wellbeing; however, some participants did report using maladaptive (avoidance) coping strategies (e.g., *“Alcohol, drugs, hookers, extreme lifestyle choices, pushing the envelope, pretty much sums up front line staffs extracurricular activities.....and no or very little social life, with questionable relationships, that lead into very dark seamy shady areas of society.”* Correctional Services, 8454). Many participants reported mental health challenges associated with occupation related stressors and described using healthy activities used to manage the associated stress. Across PSP professions, 21 participants self-reported “doing better” after having been in poor mental health and then undergoing a change or intervention, and now “doing much better” as a function of using adaptive coping strategies. Participants generally reported using three primary adaptive coping strategies: education, self-reliance, and treatment. 

### 3.1. Coping through Education

Education as an adaptive coping strategy (i.e., learning about the causes and consequences of mental health challenges) was reported as helpful for reducing internalized stigma about work-related PTSD or other mental disorders. Education was described as either personally motivated or assisted by others, and included learning about common causes, consequences, and treatments for mental disorders. Learning about the causes of mental disorders was described as particularly helpful for coping:

*Please let people know that mental health issues can be hereditary or environmental, it is not a person’s fault. They are not weak if they ask for help, it shows rather that they are strong enough to realize that something is wrong. Give them support, not pity or cast them out. It may make the difference between giving them the strength to go on, or to just give up and succumb.*

*(Correctional Services, female, 1679)*

Learning about the hereditary or environmental elements of mental disorders was one way that this PSP was able to positively reframe the negative thoughts and views she held about herself (e.g., weak vs. not weak). Other participants reported that learning about how PPTE or other stressors can impact the human brain helped them shift away from feeling isolated and at fault for their mental health challenges. The same participants reported physical, psychological, and behavioural symptoms of exposures to work-related stressors as being tied to their work environment. Participants reported that understanding how PTSD is associated with alterations in the brain had been lifesaving, by facilitating their openness to developing coping skills for managing stress:

*And I firmly believe in education and training if you know how your brain work and what happens under stress I think it is half the battle. We never have and still don’t have any formal programs in place.*

*(Municipal Police, male, 6779)*

The desire to learn about mental disorders often came about as a “last resort,” when attempts to function day-to-day became overwhelming:

*I was injured (OSI) in an extremely toxic and emotionally violent workplace. This was a daily occurrence over a 14 month period. Of course I was ashamed and it was hard to address/explain because it wasn’t caused by some sort of physical violence or threat of death etc. After 5 years I finally addressed it with professionals but it had already had a negative affect on my professional and personal life. I was diagnosed with MDD [Major Depressive Disorder] and Anxiety caused by violence and harassment in the workplace. More of a moral injury. Through my own research and reading and professional assistance I now have strategies in place to move on an[d] enjoy all life have to offer. I speak of my experience to others and provide insight into how one can overcome this type of injury.*

*(RCMP, male, 1628)*

Some PSP participants reported using problem-oriented adaptive coping to manage the stress and stigma of living with mental health disorder(s). Exploring and understanding the sources of their mental health challenges allowed some PSP to conceptualize mental health in ways that enabled them to reduce the negative impacts.

### 3.2. Coping through Self-Reliance

Adaptive coping strategies that relied on ‘self’ were typically a combination of problem-oriented or emotion-focused practices. Some participants disclosed using self-reflection to recognize behavioural changes that positively impacted their wellbeing. Participants who reported using such strategies may have also used other strategies in a trial-and-error fashion that were less effective, and decided to only report on strategies perceived as most effective. Some participants did note that changing long-standing coping strategies was very difficult. 

PSP participants reported changes in alcohol consumption and sleep as a result of external factors (e.g., impositions by employers) as particularly beneficial. PSP careers are typically characterized by non-traditional work hours, which many of the respondents implicated as a key causal factor for their mental health challenges. PSP reported that adapting their work hours resulted in routine rest and sleep cycles. Participants who reported successful changes after their sleep improved had typically been given a different shift rotation (e.g., changes in work structure) that had supported their success. For example, a male municipal police officer reported the following change in wellbeing:

*Working front line police work (uniformed patrol) tends to take a toll on you more so than where I am currently working. The shift work of working both days and nights tended to wreck my sleeping habits more and affected me more mentally I would say. I am currently in a better spot where I have gotten better with my sleeping habits as well as feeling better.*

*(Municipal Police, male, 12)*

The impact of an irregular sleep was a common reason that PSP cited to motivate themselves to change:

*I know that my answers to a lot of the work related questions would have been different 3 years ago when I [was] working shifts, my sleep was a big issue in my life and was affecting me personally and professionally. After 16 years of shift work I was able to get into a day shift job where I am currently and my work stress and home stress has gone way down.*

*(Municipal Police, male, 1023)*

Decreased alcohol consumption was reported as a behavioural change strategy to support coping by participants, although less frequently than changes in sleep patterns. PSP who consumed alcohol, but did not report addiction concerns, described reducing consumption particularly after their shifts. Participants described understanding why colleagues might consume alcohol after a shift, but for themselves chose to use a different coping strategy. Some PSP reported being in recovery from alcoholism, like the male RCMP officer who reported that, *“I have been sober for 20 years but the alcoholism is still there”* (6445), or the Correctional Services employee who reported:

*…I had a drinking problem… I answered “0” to drinks consumed per week at the present time, compared to over 80 per week about 7 months ago! Also, I used to avoid every social/ unfamiliar/ uncomfortable situation, whereas now, I face them head on.*

*(male, 8916)*

The PSP participants above reported taking control of their behaviours and making intentional changes to their coping strategies as part of improving their wellbeing. Such efforts included engaging in processes to recover from alcoholism. Adaptive coping was particularly evidenced in the problem-oriented response where a participant changed their behaviours, facing social situations “head on” rather than using maladaptive (avoidance) coping actions, which was associated with improvements in their wellbeing.

Participants also reported spending time alone as a coping strategy. Time alone was described in different ways, with some PSP describing themselves as introverts who value “quiet time”:

*I am an introvert and being around people causes me more stress than being alone does. I deal with stress by ensuring I have enough quiet time alone. Please don’t forget that some of us are introverts and as such, being around people drains energy. Extroverts need to be around people to recharge. I need to be alone to recharge.*

*(RCMP, female, 7260)*

Some participants cited the need to engage in singular activities as a means to rejuvenate from a professional life of being around people, such as finding solace in the outdoors:

*I am an enthusiastic trail runner and most weeks I get out at least twice to run trails and cross country through beautiful landscape for 2 or more hours each time... Along with being physically active, time spent in the natural world is essential for mental health.*

*(Municipal Police, 1456)*

The nuances of participant coping strategies were diverse, but consistently included valuing time alone to sort out their own needs and ‘recharge.’ Several participants reported valuing being outdoors or engaging in solo activities such as yoga, running, or mindfulness. The solo coping activities appear more emotionally oriented than problem-focused, allowing time for reflection and self-care.

### 3.3. Coping through Treatment

Seeking out and undergoing treatment to improve mental health was an adaptive coping strategy reported across occupations by male and female PSP. Participants reported seeking pharmaceutical interventions or psychotherapy mental health interventions (e.g., psychologist). Pharmaceutical interventions were used either in isolation or in combination with psychotherapy, and included sleeping aids, over the counter medications, anti-anxiety or anti-depressants, and medicinal marijuana. Over the counter medications were reportedly used as recommended, often for pain or headaches and other types of common health challenges. For example, a male paramedic explained that *“regarding pain... over-[the-]counter medication for pain—I use them daily within reason or as stated on recommended dose (e.g., Aleve or Tylenol) for arthritic or muscle/body pain”* (3481). Other PSP described using prescription drugs to assist with sleep, including when sleep was hindered by PPTE exposures:

*I had an experience where I … could have been killed. It took about 2 weeks before I realized I was having extreme anger issues and would have put the public and myself at risk. Self-awareness is key and I went and sought help and felt better afterwards. For some reason, even when nothing is bothering me I have random panic attacks while lying in bed about to fall asleep. I have been using sleep aids (Zopiclone) to assist in managing shift work sleep schedules since I started this job and to ensure I get a good night sleep even on days I am not working on occasion. Sleep has been the best helper at regulating mood and energy. Luckily I work for a Police Service that recognizes and has many support resources for employees to access should they need it.*

*(Municipal Police, male, 2693).*

The police service member’s words reveal how PPTE can negatively impact sleep habits, and how problem-oriented coping strategies can improve wellbeing. Beyond traditional pharmaceuticals that serve as sleep aids, marijuana was used by some PSP to help with sleep:

*My answers reflect me prior to medicating. My medication (~1.5g / day) reduces the reactions I have and increases my ability to sleep; but in no way is it a cure-all. Considering ALL the medications they put me on; Medical Marijuana is the only one that truly helps and without any negative side-effects. Since starting this medication I have also ceased having to need any of the other pharmaceuticals that I was previously prescribed.*

*(Firefighter, male, 3019).*

Medicinal marijuana was prescribed to PSP who struggled with sleep difficulties related to work-acquired PTSD: “I was suffering silently and not knowing I had PTSD. A doctor prescribed medical marijuana for my sleep disorders and almost instantly my PTSD symptoms disappeared” (Firefighter, male, 5081). The above firefighter descriptions echo other participants who emphasized the benefits of improved sleep for wellbeing. Participants justified marijuana use based on effectiveness, but did not similarly justify other treatments (e.g., working with a psychologist). For example, one PSP said: “But the best thing I responded to is pot; as long as it’s used in moderation. I never used pot before 18 but as an adult it has become an effective medicine” (Firefighter, male, 7253). The text echoes that from others in providing a rationale, an attempt to legitimize medical use of marijuana. Participants who disclosed use of marijuana were largely working in firefighting, clarified the use was not recreational, and explicated the benefits for symptom management. The current data were collected before legalization of cannabis, which may explain some of the emphasis placed on medicinal use of marijuana; in any case, additional research appears warranted. 

### 3.4. Coping through Psychotherapy 

PSP who engaged in sessions with a mental health professional (e.g., a psychologist) were most likely to report doing ‘better now’ after treatment. Some PSP combined both prescription drugs (e.g., anti-anxiety or anti-depressants) with therapy and reported positive outcomes:

*6 months ago my panic/anxiety was severe to the point I would lose my vision or be unable to move if I was stimulated by loud noises, or the wrong sound or smell. I have made significant progress because of mental health professionals, medication and family support to where I lead a fairly normal life now. I’m one of the lucky ones who had access to what they needed. Thank you for undertaking this survey. *

*(Paramedic, male, 3713)*

The paramedic’s words specify the combination of strategies he found helpful for addressing his mental health symptoms as well as highlighting the pervasive treatment barriers facing PSP. Such experiences were reported by respondents who felt psychotherapy was insufficient, unavailable, or their wellbeing was not prioritized. Further, treatment-seeking participants often reported the processes created potentially problematic experiences:

*Also, I would be more optimistic about professional therapy if only Alberta WCB would cover such expenses. After I completed their “TPI level 3 Program” I was told “this is as good as you will be. Now learn to deal with it.” I was then instructed that “I was done with therapy. If I needed more I was to go to Alberta Mental Health.”*

*(Firefighter, male, 3019).*

*I am doing a lot better now, but I also received help and talked about what was going on. There was a long time when I felt alone. I hid what was going on in my life from my wife and coworkers. I was scared what people would think about me, including my wife. My anxiety was crippling, to the point where I was scared to leave my house, there was a time when I was scared to drive. I felt trapped and couldn’t find a reasonable way out.*

*(Peace officer, male, 1650).*

In the former excerpt, the firefighter reveals having sought treatment as a coping strategy but experiencing several barriers to access. The quote reiterates a common theme among PSP who reported doing better after treatment but having experienced several barriers during their first attempts to seek professional care. Several PSP also reported psychotherapy as a very effective coping strategy but described their organization’s return-to-work practices as inadequate.

*I had mental health issue previously with my organization and received counselling from a psychologist and it had helped me deal with what I was going through at this time. I had taken some type of test that indicated I had mild Post Traumatic Stress Disorder, but unsure how they rate PTSD maybe in future there can be more information to patients to understand the levels. Also once I went back to work my organization didn’t do much follow up to insure that I was adjusting back at work it was like they were more concerned to get me back to work than to help me with my mental health. If you want to really help persons who have mental health injuries something has to be done to insure that first responders are cared for and the medical staff are there for the wellbeing of the patient not the employer.*

*(RCMP, male, 5910).*

PSP appear to recognize treatment as a beneficial problem-oriented coping strategy that is impeded by accessibility and return-to-work processes. Participating PSP appeared to believe the impediments to accessing evidence-based treatments are the employer’s responsibility. Perceiving treatment as difficult to access can inhibit PSP in using an important adaptive coping strategy (see Ricciardelli et al., 2018 for a discussion of the structural stigma around treatment seeking for PSP). PSP who reported doing better tended to attribute their success to treatment, specifically psychotherapy, either alone or in combination with other interventions. PSP who reported success also reported being in a better place and able to actively reflect on no longer managing active symptoms. Several participants provided a comparative perspective, describing how their answers to the current survey would have been “different” had they completed the survey while struggling with more intense symptoms of a mental health disorder. Participants often reported feeling “recovered” and not in “recovery”:

*I was diagnosed/treated regarding PTSD 3 months or so ago. It has made answering the questions difficult, because I was conflicted with before and after type problems in choosing my answers. I am also a combat vet, before I got into the emergency services. My PTSD is service related, and thought to be compounded by my emergency service. I have been hiding all of this for 29 years.*

*(Firefighter, male, 912).*

*I was diagnosed with PTSD in June 2016 following a traumatic call for service with extensive and devastating injuries to patient. Early access to psychologist and EMDR cognitive therapy most likely saved my career and marriage. I am back at work following nearly 4 month absence working again in my full capacity.*

*(Paramedic, male, 8126).*

*I am proud to state that I am a survivor of depression, anxiety and subsequently the diagnosis of PTSD and I know that it can be successfully treated and ultimately overcome. The medical system in its many outreaches has benefited me without a doubt.*

*(Correctional Services, male, 7096).*

The PSP participants above reported doing better at the time they completed the survey relative to their history of mental health challenges. Relatedly, some PSP reported still struggling with mental health challenges, being in treatment, and being on the pathway to recovery:

*I actively am receiving treatment for my condition, I have good and bad days, I know I will live with this for the rest of my life but after three years in therapy, I am gaining ownership of my ghosts and monsters and not the other way around!! I will win. Thank you and God bless.*

*(Paramedic, male, 8344).*

PSP participants described various ways of accessing and using pharmacological (e.g., sleeping pills, marijuana) and psychotherapeutic interventions to help them along their path towards recovery, almost always emphasizing important supports from co-workers, families, and friends. Participants reported diverse current mental health states and experiences, and described using an array of treatments that helped support their recovery processes. The treatments participants described complimented their adaptive coping strategies which focused on education and self-reliance. 

## 4. Discussion

PSP are exposed to PPTE at exceptionally high frequencies [1] and appear to experience mental disorders at rates much higher than the average Canadian citizen [3]. Arble, Daugherty, and Arnetz, 2018 present a model of PSP (using the term first responder) coping that recognizes PSP “could engage in numerous coping strategies that ultimately translated to greater overall well-being” [22] (p. 163). Use of personal coping strategies must be understood as a dynamic process, with the effectiveness of each strategy being heavily contextually-dependent [4,29] and interacting with gender, role, and type of work performed [19], as well as time since and magnitude of any given PPTE [26]. 

The current study was designed to improve our understanding of the coping strategies used by PSP who self-reported as “doing better”, therein indicating they experienced or were experiencing improvements in their mental health and wellbeing. A sense of personal agency appeared centrally associated with improvements among participants who changed their lives in ways that improved their mental health (e.g., limiting alcohol use, changing sleep patterns). Shifts in participant perceptions of well-being, work, family, and life also appeared to be important for improving mental health, which is consistent with other research on personal mental health recovery processes [37,38,39,40]. Personal recovery has been described as improvements in daily functioning that extend from symptom reductions [37], with a focus on having meaning in life and personal transformation [37]. The current results also emphasize that PSP mental health may benefit from engaging external factors, such as education, psychotherapy, and social supports. 

Learning about the causes and consequences of mental disorders were one coping method described by PSP participants. There is evidence that psychoeducation benefits civilian populations [41,42]; specifically, cross-sectional survey data from 192 outpatient clients with schizophrenia underscored the benefits of learning about the mental disorder and strategies to enhance their daily lives. There is also evidence of an inverse relationship between the desire to learn more about a disorder and the length of time spent experiencing the disorder [41]. Research exploring the desire, benefits, and drawbacks of developing and delivering psychoeducation to persons with mental health challenges may be important for designing PSP-specific educational resources. A meta-analysis on 36 studies found small to moderate program effect sizes for reductions in PTSI symptoms in public safety and frontline healthcare professionals and for promoting measures of well-being [21].

Educational resources designed for PSP typically focus on skills training designed to promote positive adaptation to stressors and help PSP cope with PPTE [42,43]. There may be additive benefits from providing disorder-specific psychoeducation to support adaptive coping; however, the research on psychoeducation and mental health remains mixed. In a randomized control trial Anderson, Vaughan, and Mills (2017) found evidence that paramedic students who participated in an online course introducing various adaptive coping strategies and stress recognition strategies improved short-term personal resilience among primary care paramedic students [42]. Similarly, Arnetz and colleagues (2009) found evidence that training early career police officers on stress and performance during a simulated PPTE improved mood, reduced cardiac reactivity, and improved behavioral performance (verbal and physical) when compared to a control group [44]. Mindfulness-Based Resilience Training also appears to partially mediate the relationship between mindfulness and burnout [43]. 

Cross-sectional evidence indicates that that participating in some training programs (e.g., Mental Health First Aid, Road to Mental Readiness, Critical Incident Stress Management) is associated with slightly lower probabilities of screening positive for some mental health disorders [45]. In contrast, a longitudinal assessment of the Road to Mental Readiness (R2MR) training with police evidenced no statistically significant changes in mental health symptoms, resilience, or work engagement, but did evidence small, statistically significant, reductions in stigma at post-training [46]. Another study of R2MR also evidenced small, but statistically significant, effect sizes for stigma reduction and self-perceived resiliency increases at three months post-training [47]. A randomized control trial, found no evidence that a resilience-focused training program delivered to military recruits in the United Kingdom impacted symptoms of PTSD, common mental health disorders, or alcohol use [48]. Despite the mixed evidence, psychoeducation appears to be a relatively inexpensive intervention that can reduce stigma and facilitate treatment-seeking behaviours.

Having access to evidence-based mental health treatment options that can be tailored for PSP may be integral to sustaining or recovering good mental health. PSP, their leaders, and their health care providers may all benefit from knowing repeated PPTE exposures can have a substantial negative impact on PSP mental health. Rutkow, Gable, and Links (2011) argued that society has a legal and ethical obligation to ensure policies are in place that will support PSP in receiving adequate and quality health care [49]. They proposed a multi-faceted approach to address some of the current challenges in providing adequate care for PSP [49]. The approach included recommending regular mental health screening for all PSP, to ensure timely diagnoses and treatment [49]. The approach also included revising worker’s compensation program policies to expedite PSP mental health claims, which would allow for faster access to evidence-based mental health care [49].

PSP participants identified psychotherapy as beneficial, alone or in conjunction with other interventions. A study focused on providing individual sessions from doctoral-trained therapists to support overall PSP wellness evidenced significantly higher wellness scores for treatment participants relative to a control group [50]. Evidence-based psychotherapy provided by a qualified mental health professional appears to be effective, but increasing attention is also being paid to peer-to-peer supports as part of stepped-care models. The shift in focus may be due to relative availability of providers and the workplace culture that exists within PSP organizations. There is no dispute that individuals who work as PSP are part of cultures characterized by particular experiences, language, values, and world views [51]; accordingly, PSP may view mental health professionals as “outsiders” [52]. Researchers have indicated PSP prefer to speak with peers [53] rather than mental health professionals [45]; therefore, interventions based on peer relationships may help PSP to relate, to receive empathy, and to facilitate access other levels of care for treatment [54]. 

Research with non-PSP populations has evidenced promising preliminary results for supporting groups and organizations led by peers who share occupational backgrounds in support of recovery processes [55]. In the United States, “Stay Connected” was an initiative to support PSP after the 9/11 terrorist attacks [56]. Participants reported being very satisfied with the mental health supports provided through “Stay Connected” [57]. The peer-directed program REACT (i.e., React, Evaluate, Advocate, Coordinate, Track) was created specifically to address and support PSP mental health [58]. There were also three crisis intervention programs developed, deployed, and examined for law enforcement officers who responded to 9/11 and Hurricane Katrina [52]: psychological first aid, critical incident stress management, and the Federal Emergency Management Association/Substance Abuse Crisis Counselling Program. All three programs were designed to include peer support [52]. Overall, the propagation of peer support programs for PSP indicates a pervasive perception that peers can be critical for supporting mental health.

The PSP participants emphasized how changing their sleep habits improved their mental health, which is consistent with numerous research results underscoring the central role of sleep for positive mental health and wellbeing [37,38,39]. The PSP employer and the PSP employee need to share responsibility for facilitating and maintaining positive sleep hygiene in support of mental health. Sleep is essential for coping, but all problem- focused, and emotion-oriented coping strategies may be interdependent and versatile.

The current study contributes to our knowledge of coping strategies that PSP self-report as helpful for mitigating mental health challenges; however, there are several limitations that caveat generalizability of the results and offer directions for future research. The current qualitative research results should not be generalized beyond the current study participants. The survey question that participants answered was very broad in nature and did not address the issue of coping directly, which may have limited the data collection. Responses used in the current study were volunteered, which means the results only capture participants who chose to share information on their coping; nevertheless, that caveat serves as evidence of the importance PSP placed on showcasing the coping strategies they found beneficial. Future researchers should attempt to replicate the current results using larger, randomly selected and demographically stratified samples assessed with multimodal data collection methods.

## 5. Conclusions

Problem-focused and emotion-oriented approach coping strategies that deliberately analyze and process PPTE are often perceived as being healthier than avoidant coping strategies [22,30]. Approach coping strategies have been associated with adaptive coping and improved outcomes after PPTE exposures, including a more positive outlook and fewer intrusive thoughts [26]. In the current study we took a novel approach to examining coping strategies employed by PSP who self-reported feeling better, to some degree, and experiencing improved mental health and wellbeing.

Understanding how PSP cope with stress is critical for ensuring their wellbeing. The current results describe PSP as often simultaneously using diverse coping strategies to manage their exposures to occupational stressors. Consistent with previous quantitative data [19,22,26], the current qualitative data evidence adaptive coping strategies as involving education, self-reliance, and evidence-based treatment.

Participating PSP expressed a desire for their employers to take more interest in their mental health before, during, and after PPTE exposures, which is consistent with adaptive and proactive coping strategies. Psychoeducational programs in the workplace may have as much as a nine-to-one return on investment [59] by helping participants learn healthy proactive coping strategies, recognize developing disorders, become aware of treatment and support options, and develop skills to provide mental health first aid [60]. Accordingly, PSP employers may benefit from initiating evidence-informed psychoeducational courses and support programs to help PSP manage stress as well as minimize the frequency and impact of mental health injuries.

## Figures and Tables

**Table 1:** Number of PSP participants by occupation.

PSP Occupational Group	TOTAL
Call centre dispatch/operator	26
Canadian Border Services	20
Other (e.g., coast guard, coroner, administrative)	12
Correctional work, administrative	21
Correctional work, operational	92
Pre-medicine (paramedic, EMR, EMT)	146
Firefighter	103
Other fire (fire/paramedic, volunteer, search and rescue)	25
Municipal police	142
Provincial police	30
RCMP	208
Other police (transit, special constable)	5
Not specified	8

**Table 2:** Primary, secondary, and tertiary coding themes.

Primary Theme	Secondary Theme	Tertiary Theme
“Better now” but struggled in the past	Sleeping better	
	Reduce alcohol consumption	
	Seeking treatment and intervention	
Social Supports	Family and friends	PSP spouse
	Pets	Peers in similar profession
	Peer Support	Concern about lack of confidentiality
		Feeling needs are ignored by management
Treatment	Counseling or psychology	Sleeping pills
	Medical interventions	Medicinal marijuana
		Over the counter meds
		Anxiety meds
		Stress leave
Education	Learning how the brain works	
	Hereditary nature of mental illness	
	Talking to people	
	Educating others	
Other	Being in nature	
	Spending time on their own	
	Recharging by being around people	
	Negative behaviours (e.g., drinking alcohol)	

## Data Availability

Data is only available on request due to privacy/ethical restrictions. The data are not publicly available due to their containing information that could compromise the privacy of research participants.
